# Editorial: Optical imaging in neuroscience and brain disease

**DOI:** 10.3389/fnins.2023.1192863

**Published:** 2023-04-04

**Authors:** Guanglei Zhang, Xibo Ma, Wenjian Qin, Mengyu Jia, Maomao Chen

**Affiliations:** ^1^Beijing Advanced Innovation Center for Biomedical Engineering, School of Biological Science and Medical Engineering, Beihang University, Beijing, China; ^2^State Key Laboratory of Multimodal Artificial Intelligence Systems, Institute of Automation, Chinese Academy of Sciences (CAS), Beijing, China; ^3^Shenzhen Institute of Advanced Technology, Chinese Academy of Sciences (CAS), Shenzhen, China; ^4^School of Precision Instruments and Optoelectronics Engineering, Tianjin University, Tianjin, China; ^5^Department of Medicine, University of Pittsburgh, Pittsburgh, PA, United States

**Keywords:** brain disease, optical imaging, neuroscience, biological applications, imaging systems, imaging methods

## Preface

Optical imaging has developed rapidly in recent years owing to its high sensitivity, strong specificity, and fast imaging speed (Weissleder, 2006). Optical microscopy, including confocal microscopy (CM; Elliott, 2020), light-sheet microscopy (LSM; Hillman et al., 2019), multi-photon microscopy (MPM; Diaspro et al., 2006), super-resolution microscopy (SRM; Schermelleh et al., 2019), and photoacoustic microscopy (PAM; Yao and Wang, 2013), etc., provides nanometer to micrometer spatial resolution and microsecond temporal resolution, which allows the studies of neuroscience and brain disease at the cellular or molecular level. Optical tomography on the other hand, including bioluminescence tomography (BLT; Zhang et al., 2022), fluorescence molecular tomography (FMT; Zhang et al., 2021), x-ray luminescence tomography (XLT; Zhang et al., 2018), Cerenkov luminescence tomography (CLT; Li et al., 2010), photoacoustic tomography (PAT; Xia et al., 2014), and diffusion optical tomography (DOT; Yoo et al., 2020), etc., achieves the penetration depth of a few centimeters, making it possible to study the neuroscience and brain disease *in vivo*. These optical imaging technologies have enormously expanded the research of neuroscience and brain disease across multiple scales, from the molecular and cellular level to the whole brain level. The objective of this Research Topic is to highlight the cutting-edge methods (e.g., methodological improvements, new algorithms, validation studies, etc.) and novel applications (e.g., biological applications, pre-clinical applications, clinical applications, etc.) of two-dimensional (2D) and three-dimensional (3D) optical imaging technologies used to carry out various studies of neuroscience and brain disease.

## Editorials

Zhang et al. mainly introduced a new approach to treat Alzheimer's disease by using the synergistic photobiomodulation of 808 nm and 1,064 nm lasers to reduce the neurotoxicity of β-amyloid protein in an *in vitro* Alzheimer's disease model. To better understand the mechanism of action of laser irradiation, the authors performed a series of cellular and molecular biology experiments, including cell proliferation and apoptosis analysis, fluorescent staining, Western blotting and real-time fluorescent quantitative PCR. The authors found through experimental studies that this synergistic effect can significantly reduce the neurotoxicity of β-amyloid protein and promote the growth and survival of nerve cells. The study provides a new idea for using photobiomodulation to treat Alzheimer's disease. The findings of this study are of great significance for the treatment and prevention of Alzheimer's disease.

Debnath et al. explored the effect of neuromodulatory signaling pathways on the magnitude of calcium transients and the implications of this effect on the increase or decrease in neuronal depolarization. The study used calcium imaging to measure the magnitude of calcium transients within neurons and simulated different neuromodulatory signaling pathways by drug treatment. The findings suggest that different neuromodulatory signaling pathways respond differently to transient calcium amplitude, with some signaling pathways increasing transient calcium amplitude and others decreasing it. In addition, increases or decreases in the amplitude of calcium transients can also have different effects on neuronal depolarization, depending on the type and state of the neuron. These research results provide new ideas and approaches for understanding the mechanism of intra-neuronal signaling and neuromodulation to be used for the treatment of neurological diseases.

Senthilvelan et al. performed a non-invasive assessment of hemoglobin parameters in patients with dural arteriovenous fistula (dAVF) by using near-infrared spectroscopy (fNIRS) technology. The study used a new algorithm to analyze fNIRS data to assess parameters such as blood oxygen and hemoglobin concentrations in dAVF patients. The purpose of this study was to explore a new method to evaluate the disease progression of dAVF patients, so as to help patients understand the changes and development trends of the disease more accurately, thus better guide treatment and prognosis assessment. The results of this study show that fNIRS technology can provide real-time blood oxygen and hemoglobin concentration information, and has the advantages of being non-invasive, non-radioactive, and thus has great promise for application.

Wang et al. proposed a novel quantitative optical coherence microscopy (QOCM) method for high-resolution imaging and quantitative analysis of neuronal morphology in the human medial temporal lobe cortex. This study uses the QOCM method to study the morphological structure of human brain neurons to help better understand brain functions and diseases. The results of this study demonstrate that QOCM can effectively image and quantitatively analyze the morphological structure of neurons in the human medial temporal lobe cortex, including characteristics such as neuron size, shape, branching, and connectivity. The results of this research provide new tools and methods for neuroscience research, which can help better understand the structure and function of the brain and provide new ideas and strategies for the diagnosis and treatment of neurological diseases.

Chen et al. proposed a weakly supervised learning method to analyze the distribution of amyloid-beta (Aβ) plaques in the whole rat brain. The proposed algorithm is based on a weakly supervised learning approach that enables automatic classification and analysis of large amounts of unlabeled data by using a small amount of labeled data to train the model. The purpose of this study is to explore a new method to analyze the distribution of Aβ plaques in the rat brain to help better understand the role and influence of Aβ plaques in the nervous system. The results of this study show that the weakly supervised learning method can effectively analyze the distribution of Aβ plaques in the rat brain, which has great promise for application and can provide new ideas and methods for neuroscience research.

## Author contributions

GZ wrote the text. XM, WQ, MJ, and MC were co-editors of the Research Topic and edited the text. All authors contributed to the article and approved the submitted version.
